# Phase transitions in the classical simulability of open quantum systems

**DOI:** 10.1038/s41598-023-35336-9

**Published:** 2023-05-31

**Authors:** F. Azad, A. Hallam, J. Morley, A. G. Green

**Affiliations:** 1 London Centre for Nanotechnology, University College London, Gordon St., London, WC1H 0AH UK; 2 School of Physics and Astronomy, University of Leeds, Leeds, LS2 9JT UK

**Keywords:** Mathematics and computing, Physics

## Abstract

We introduce a Langevin unravelling of the density matrix evolution of an open quantum system over matrix product states, which we term the time-dependent variational principle-Langevin equation. This allows the study of entanglement dynamics as a function of both temperature and coupling to the environment. As the strength of coupling to and temperature of the environment is increased, we find a transition where the entanglement of the individual trajectories saturates, permitting a classical simulation of the system for all times. This is the Hamiltonian open system counterpart of the saturation in entanglement found in random circuits with projective or weak measurements. If a system is open, there is a limit to the advantage in simulating its behaviour on a quantum computer, even when that evolution harbours important quantum effects. Moreover, if a quantum simulator is in this phase, it cannot simulate with quantum advantage.

The term classical is applied to quantum systems in at least two different ways. On the one hand, if a closed quantum system is in a weakly-entangled state, it may be considered classical as long as the entanglement remains low. In this limit, the equations of motion of the system are termed semi-classical. On the other hand, an open system behaves classically once the coupling to the environment has caused dephasing of the off-diagonal elements of the density matrix. These two limits occur on very different timescales—the semi-classical limit at early times and the dephasing limit at late times.

Can these views be reconciled and a classical description developed that works from the earliest to the latest times? Recent insights have made steps towards such an understanding for open systems. The transition in entanglement growth in random circuits as a function of the rate of projective or weak measurement allows a classical, weakly-entangled description of the system for all times^1–7^. Both are cousins to the quantum Zeno effect, by which frequent measurement in a channel impedes transitions in that channel^8^. The nature of the many-body transition has been studied extensively^9–19^ and similar analyses extended to measurement-induced transitions in open Hamiltonian systems^20–23^. These later cases are closely related to a transition in classical describability as a function of coupling to the environment, which we consider.

Our approach is to use a variational parametrisation of trajectories obtained by unravelling the evolution of an open system density matrix. The equations of motion of each trajectory can be considered a Langevin extension of the time-dependent variational principle (TDVP) — which we derive and present here for the first time. For closed systems, the TDVP equations constitute a semi-classical limit; they correspond to classical Hamilton equations of motion on the variational manifold^24,25^. As the entanglement grows during the Hamiltonian evolution, the TDVP equations break down as a larger and larger variational manifold is required in order to capture the state and its dynamics. In this sense, the semi-classical description is confined to early times. In our stochastic TDVP Langevin equation, we find thresholds in the dynamics of individual trajectories as a function of coupling to and temperature of the environment, whereby the entanglement saturates at a low value, and a low-bond order matrix product state description gives high fidelity for all time. This *quantum Zeno phase*^2^ constitutes a transition in the classical describability of the open quantum system: the low bond order TDVP Langevin equation is an effective semi-classical description that works for all time.

## A TDVP Langevin equation

**Figure 1:**
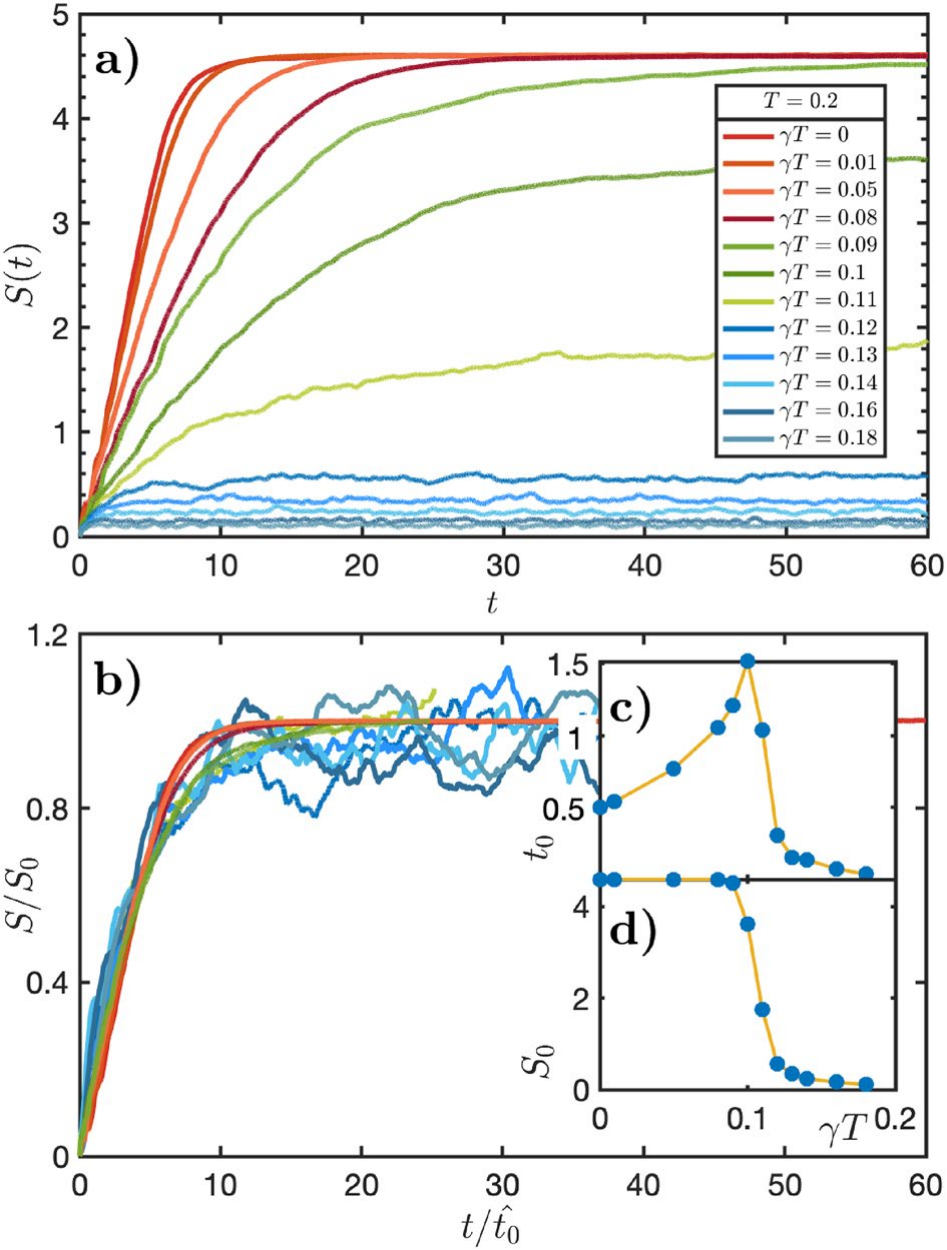
*Basic Properties of MPS Langevin Evolution:* The TDVP Langevin equation, Eq. (1) describes the evolution of the density matrix through an ensemble of stochastic pure-state trajectories. (**a**) *Half-chain* *von Neumann entropy as a function of time* averaged over 70 trajectories at different coupling strengths for chains of length 16. Temperature is fixed at $T=0.2$, while friction, $\gamma$ (and therefore noise, $\gamma T$) is varied. Simulations were carried out at bond dimension $D=128$. Three different regimes of behaviour are apparent: an initial transient, followed by an approximately linear-in-*t* growth, and finally a saturation. At low values of $\gamma$ this saturation is determined by the variational approximation. At high values it is determined intrinsically by the interplay of the Hamiltonian and the dissipative bath. It signals whether a lower bond-dimension (hence less computationally intensive) simulation suffices in to capture the entropy dynamics. (**b**) *Scaling collapse* of the data shown in a). Amplitudes and timescales are rescaled by factors of $S_0$ and $t_0$, respectively (Fits to $S(t) = S_0 \tanh (t/t_0)$ are used as a guide to this rescaling). These are plotted *versus* $\gamma T$ in the insets (**c**) and (**d**), which show clear evidence of a transition at around $\gamma T \approx 0.11$, beyond which the saturation entanglement is determined by balance between entangling effects of the Hamiltonian and environmental dissipation.

Langevin equations describe the motion of a system coupled to an environment (or alternatively the motion of slow collective degrees of freedom in an effective bath described by the faster degrees of freedom^26^) by adding noise and friction terms to the basic equations of motion of the system. If the environmental degrees of freedom are in thermal equilibrium, the friction and noise satisfy a fluctuation-dissipation relation. Applied to quantum systems, the Schrödinger equation provides the basic equations of motion. The ensemble of the resulting stochastic Schrödinger trajectories recovers the density matrix evolution and is said to be an unravelling of it.

*The TDVP Langevin equation* can be written in its Markovian limit as:

1 $$\begin{aligned} \langle \partial _i \psi | \partial _j \psi \rangle \dot{X}_j= & {} -i \langle \partial _i \psi | \hat{H} | \psi \rangle -i \sum _n \langle \partial _i \psi | \hat{F}_n | \psi \rangle \eta (t) \nonumber \\{} & {} -i \sum _n \gamma \frac{\langle \psi | \hat{F}_n | \psi \rangle }{dt} \langle \partial _i \psi | \hat{F}_n | \psi \rangle . \end{aligned}$$

The terms on the left-hand side and the first term on the right constitute the conventional time-dependent variational principle (TDVP) equations^24^ projecting the Schrödinger equation onto a variational manifold. The second and third terms on the right are the noise and friction due to coupling to the environment. $\hat{F}_n$ are the operators by which the system is coupled to the bath displacement operators. We generally assume these to be spatially local. For spin-half chains they are given by the *x*, *y* and $z-$components of the spin operators on each site, each of which couples to a separate bath. The bath is described as a collection of harmonic oscillators and the noise-correlator is determined by the spectrum of oscillators and the temperature of the bath; $\langle \langle \eta (t) \eta (t') \rangle \rangle =2 \gamma T \delta (t-t')$ in the combined classical and Markovian limits.

This approach to unravelling the density matrix evolution has some particularly attractive features. It can treat coupling to a finite-temperature and non-Markovian environments, expanding the relevance of environment-induced many-body Zeno transitions. The TDVP Langevin equation can be used with any variational parametrisation that permits a TDVP treatment in the absence of an environment. Consequently, it is possible to include the effects of long-range interactions in one dimension^27,28^. Furthermore, the approach lends itself particularly well for combination with stochastic TDVP evolution of neural quantum states^29–31^. Here we use one-dimensional matrix product states (MPS)^32–34^. To date, MPS techniques have been employed in the study of open systems largely by starting with the Lindblad master equation and either describing the density matrix directly as a matrix product operator^35–37^ or else unravelling its evolution over MPS representations of quantum trajectories^38–40^. These methods cannot treat finite temperature or non-Markovian environments for which alternative methods are required^41^.

*A derivation* of Eq. (1) is given in Supplementary Materials 1. We develop the Langevin equation from the Keldysh path integral for the time-evolution of the density matrix. The method follows that of Ref.^42,43^ with the modification that the Keldysh path integral is constructed over matrix product states^44^. The result adds noise and friction to the time-dependent variational principle constructed over matrix product states^24^. The same construction for other variational classes leads to a similar stochastic equation of motion, which we dub the TDVP-Langevin equation. Alternative unravellings of the Lindblad equation for the density matrix evolution over MPS^38^ apply in different circumstances of relative time and energy scales of the bath and system. We construct Eq. (1) over matrix product states using conventional methods^24^. Integration of this equation is complicated by the friction term. Naively, this requires inversion of a matrix that is proportional to the system size and dimension of the variational manifold. However, recognising that it consists of an outer product of vectors allows an efficient inversion and integration of the equations of motion. Details are given in Supplementary Materials 1 and our code is available at https://github.com/AndrewHallam/Langevin.

Thermal distributions are fixed points of the TDVP Langevin evolution. A thermal distribution is given by a Boltzmann-weighted Haar average over the variational manifold. For MPS of bond dimension *D*, this average can be performed as a Haar integral over the group *SU*(*dD*) with *d* the local Hilbert space dimension [see Supplementary Materials 1]. Figure 1 captures the dynamics of the von Neumann entanglement entropy. For temperature fixed at $T=0.2$ there is a transition in entanglement dynamics beyond a critical value, $\gamma T\approx 0.11$. The saturation entanglement becoming intrinsic to the interplay between the Hamiltonian and the dissipative bath, rather than by the choice of variational manifold. We detail these results in the following section. Similar transitions occur when friction, $\gamma$, or noise, $\gamma T$ are kept constant. This data is presented in Supplementary Materials 1.

## Open evolution of a rapidly entangling system

In the absence of coupling to a bath, TDVP equations eventually fail as the entanglement grows beyond that which can be represented on the variational manifold (TDVP equations for the thermofield purification of the density matrix may escape this fate^25^ at least as far as local observations are concerned). However, just as observed in projective measurements of random circuits, the effects of the environment may restrict the growth of entanglement. *In extremis* this might limit entanglement of individual trajectories so that they can be represented on low dimensional variational manifolds. The TDVP Langevin equation will then give a good account of the dynamics at all times, signifying a transition in its classical representability. This is our interpretation of the sequence of results presented in this section.

*The Hamiltonian* that we consider is the tilted field Ising model

2 $$\begin{aligned} \hat{H} = -\sum _i \left[ J \sigma _i^z \sigma _{i+1}^z +h \sigma ^z_i+ g \sigma ^x_i \right] , \end{aligned}$$

with $J=1$, $g=-1.05$ and $h=0.5$. With these parameters, the Hamiltonian is far from any integrable point and rapidly thermalising^45,46^.

**Figure 2:**
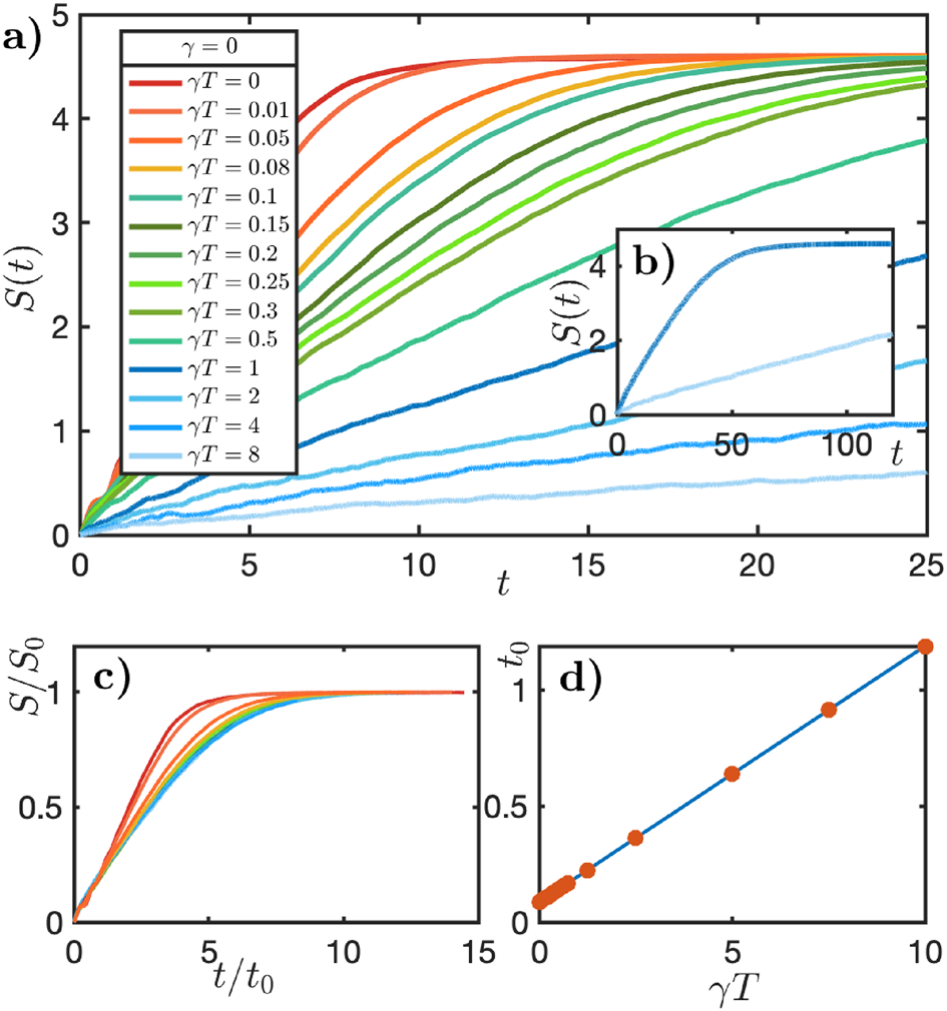
*Evolution of Entanglement at Infinite Temperature:* (**a**) Half-chain von Neumann entropy versus time for different values of $\gamma T$ with $\gamma =0$ (infinite temperature) for chains of length 16. Analogously to Fig. 1, after an initial transient, the entanglement grows linearly with time before saturating at a value determined by the MPS manifold (here with $D=160$). The rate of entanglement growth reduces with increasing noise $\gamma T$ due to dephasing effects of the bath. Unlike the finite temperature and friction case in Fig. 1 all curves saturate to the same entanglement. We anticipate that in the absence of restrictions imposed by the system size and/or choice of variational manifold the entanglement would continue to grow linearly in time. (**b**) Saturation of entanglement for higher $\gamma T$ curves illustrated by extending the simulations to $t=120$. (**c**) Rescaling the time coordinate of the data by a factor $t_0$ collapses the data onto a single curve (Fitting to a function $S(t)= S_0 \tanh {t/t_0}$ is used to extract the rescaling factors. The scaling is remarkably good beyond $\gamma T=0.15$. (**d**) Timescales extracted in this way show a linear dependence upon noise $\gamma T$.

*Infinite temperature and vanishing friction* Fig. 2a shows the variation in von Neumann entanglement across the central bond as a function of time for simulations with range of noise strengths. The simulations are carried out at bond order $D=160$, which sufficiently fully captures the Hilbert space at this system size. The broad result of these simulations is that the entanglement saturates at long times at a value determined by the system size. This is consistent with an infinite temperature final state with the maximum entanglement supported by the variational manifold. The most interesting aspect of these results is the decreasing rate of early-time entanglement growth with increasing noise strength. Crucially we do not find evidence of an intrinsic saturation of entanglement – only that dictated by the limitations of the variational approximation. Figure 2b shows the collapse of these data after rescaling time by a factor $t_0$ (obtained by fitting to the function $S(t)=S_0\tanh (t/t_0)$). The saturation entanglement $S_0\approx 4.6$ is the same for all noise strengths. Figure 2c shows the saturation time $t_0$ obtained in this way. This fitting is very good beyond $\gamma T=0.15$. Beyond this coupling strength, the dynamics are similar to random unitary circuits with conservation laws ^3,47^. Indeed, setting the external fields in Eq. (2) to zero generates the same dynamics. Figure 2d shows a clear linear scaling of $t_0$ with the strength of noise.

*Finite temperature and friction* Including both noise and friction, we see a transition into a many-body Zeno phase in which entanglement saturates. This is demonstrated in two ways; by considering the saturation of entanglement at long times and by a high fidelity between low- and high-bond order simulations at long times. This transition is evident in the entanglement entropy data for fixed $T=0.2$, shown in Fig.1, where increasing coupling strengths causes reduced saturation entanglement. This transition can also be observed in different cuts through the noise-friction ($\gamma - \gamma T$) plane, data for which is presented in the supplementary materials.

**Figure 3:**
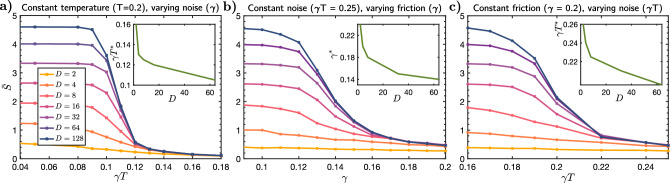
*Evolution of Saturation Entanglement at Finite Temperature:* In the main figures we show the dependence of the von Neumann entropy as a function of noise and friction: a) *versus* $\gamma T$ at fixed *T*, b) *versus* $\gamma$ at fixed $\gamma T$, c) *versus* $\gamma T$ at fixed $\gamma$. In each case, at low values of noise and friction, the saturation entanglement $\bar{S}$, is determined by the choice of variational parametrization through the bond order. As the noise and friction are increased, there is a cross-over where the saturation entanglement decreases from this value. Each bond dimension captures the saturation entanglement for a sufficiently large noise and friction. This is indicated when the entanglement begins to follow the entanglement given by the highest bond dimension simulation. At the transition, the saturation entanglement of the lower bond dimensions converges to that of the highest bond dimension. Beyond this point the saturation entanglement is determined intrinsically by the interplay between the Hamiltonian and the environment. *In each corresponding inset figure*, we have extracted critical dissipation strengths where these transitions occur as a function of bond dimension.

**Figure 4:**
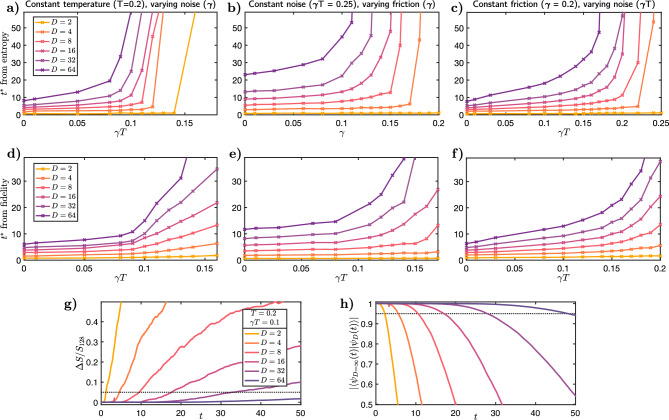
*Divergent Classical Simulation Time* Simulations carried out at a bond order *D* give a good account of the system evolution up to a time $t^*(D)$. We extract these values versus a reference $D=128$ simulation, which serves as the reference account of the system. We do this in two ways, by comparing the difference in von Neumann entanglement entropy between these states and the fidelity with this state. $t^*(D)$ is the time when the simulation with lower bond dimension deviates appreciably from the reference trajectory. The row with panels a), b), c), shows $t^*(D)$ extracted from the entropy, while panels d), e), f) shows $t^*(D)$ extracted from the fidelity. The fixed variables are split across the columns – a), d), shows varying $\gamma T$ at fixed $T=0.2$, b), e) varying $\gamma$ at fixed $\gamma T=0.25$, and c), f) varying $\gamma T$ at fixed $\gamma =0.2$. Panels g) and h) show typical evolution of entanglement and fidelity with time. These data are taken from the the trajectory with noise $\gamma T = 0.1$ which is close to the critical point for $T=0.2$. A simulation is judged to have ceased to provide a good account of the system when the trajectory deviates beyond $\epsilon =0.05$, and the time at which this occurs is $t^*(D)$. In g), this is the point where $\Delta S/S_{D=128} = |S_{D=128}-S_{D}|/S_{D=128} > \epsilon$. Analogously in h), $t^*(D)$ is the time when the fidelity is appreciably different from 1, i.e. $|\langle \psi _{D=128}(t)|\psi _{D}(t)\rangle |< 1-\epsilon$. A divergent $t^*(D)$, within either method of extraction, indicates a transition in the classical simulability of the open quantum system.

(i) *Saturating entanglement* In order to demonstrate this, we first show in Fig. 3 the long-time average of the von-Neumann entropy, $\bar{S}$. The simulations are carried out for a time 60 (in units of *J*) and averaged over the interval 50 -60. A graph showing the typical time-dependence from which such saturation values are computed is shown in Fig.1 a). For low noise and friction, the saturation is determined by the limitations of the variational manifold. Panels a), b) and c) show $\bar{S}$ as a function of $\gamma T$ at fixed $\gamma$, *T* at fixed $\gamma$, and $\gamma T$ at fixed $\gamma$, respectively. A threshold is reached for each bond dimension where it adequately captures the saturation entanglement, thus indicating a transition to increasingly classically simulatable dynamics. The transition can be seen from the point where the trajectories obtained at different bond dimensions give the same saturation entanglement. From this we can extract a critical $\gamma$ or $\gamma T$ as a function of bond dimension that we show in the insets. Note that since these data show the entanglement averaged over time 50-60, in some cases the saturation entanglement has not yet been reached. The transition is therefore expected to be slightly sharper than that shown in the figures. Compare for example Fig. 1d with Fig. 3a for example.
(ii) *Fidelity as* $t \rightarrow \infty$ We can identify an analogous transition in the fidelity of each trajectory at different bond dimensions *versus* a reference trajectory with bond dimension $D=128$. In this case, we find that beyond a critical combination of $\gamma$ or $\gamma T$, the fidelity of the state at low bond dimension remains close to 1 for long times. We expand upon this result in Fig. 4, where we identify a divergent classical simulation time. We note that the fidelity is more sensitive to the time-step as friction is increased – an issue typical of numerical integration of systems of stochastic differential equations. This makes accessing the critical point of the transition numerically intensive for the parameters and Hamiltonian we consider. The entropy is less sensitive to this.

## Limits on classical simulability

Whilst the TDVP Langevin equation cannot efficiently describe a system in the many-body Zeno phase, the question of how tight is this bound on simulation remains. Can efficient methods of classical simulation be found that extend into the region that we have identified as the many-body Zeno phase? For example, as a system approaches thermalisation, it is more efficient to use a density matrix description^49–51^, a variational representation of its purification^25^ or a neural quantum state^63^ than an unravelled description in terms of pure state trajectories. Indeed, these latter descriptions are efficient both at early times where entanglement is weak and at late times as thermalisation occurs. Nevertheless, there are good grounds to believe that our bound on classical simulability is strong.

Our reasoning is as follows: the complexity of open system quantum dynamics is not monotonic. There is a large information or complexity barrier between efficient early- and late-time descriptions in the generic case. The question of whether an efficient classical description is possible is essentially asking whether coupling to the environment suppresses this complexity barrier. When a system initially in an unentangled state is weakly coupled to a finite temperature bath, the early-time evolution is characterised by a growing complexity similar to that due to the growing entanglement in the closed system. The TDVP Langevin approach applies in this limit. Alternative approaches such as MPO representations of the density matrix or neural quantum states have similar (or higher) complexity in this limit. At late times the complexity decreases as the system approaches thermalisation, and ultimately a hydrodynamic description is appropriate.

We address the issue of classical simulability from the early time side of the complexity barrier where TDVP Langevin evolution of matrix product states is appropriate—and where its efficiency is at least as good as alternative schemes. This allows us to ascertain whether the complexity barrier has been suppressed by coupling to the environment. MPO descriptions of the density matrix, MPS descriptions of its purification or neural quantum states are efficient both in the early- and late-time limits. Such descriptions must still surmount the complexity barrier and their late-time efficiency does not help in this. Indeed, we anticipate that analyses using different variational parametrizations to determine whether the early-time growth of the complexity barrier is suppressed will yield similar results notwithstanding the issue of their late-time efficiency.

What is the origin of this complexity barrier and how high is it? The peak in complexity emerges in the balance between the entangling effects of the system’s Hamiltonian and the thermalising effects of the bath. We identify two timescales: i. the timescale at which entanglement saturates ($S_{sat} \sim L \log d$) in the absence of thermalisation $t_{sat}$, (also known as the Thouless time^52^); ii. the timescale $t_{therm}$ at which the system approaches thermalisation with the bath (this $t_{therm}$ should not be confused with internal relaxation timescale, which for brevity we assume is much longer than $t_{therm}$). On this timescale the complexity decreases to that of the reduced density matrix on the length scale of the thermal correlation length $\xi (T)$; *i.e.* an operator entanglement $\sim \xi (T) \log d$ and operator bond dimension $d^{\xi (T)}$.

The complexity barrier between early and late times depends upon the relative size of $t_{sat}$ and $t_{therm}$. The worst case is when $t_{therm} \gg t_{sat}$, this is the complexity of an arbitrary state of the system. Whereas if $t_{therm} <t_{sat}$ then the complexity barrier may be considerably less—and an efficient classical simulation possible. In this limit, as shown by the results presented here, the ballistic early growth of entanglement may also be suppressed. Moreover, a complete understanding of the thermalisation process from this complexity point of view is also lacking. A reliable estimate of the height of the complexity barrier is therefore still unavailable.

It is worth comparing this discussion with recent classical time-evolution algorithms that permit an efficient description of local observables in a closed, thermalising system valid for all time^25,53,54^—the central idea in these works is that correlations beyond a certain lengthscale never affect local observables and so may be neglected. When coupled to an external bath the explicit restriction to local observables is not required since the bath explicitly decoheres longer-range correlations. Whether an internal or external bath, the underlying principles seem rather similar.

## Discussion

This work introduces a new method to investigate the dynamics of open many-body quantum systems, the TDVP-Langevin equation. We derive this by considering an appropriate limit of the Keldysh path integral constructed over a variational manifold. We carry this out explicitly for the MPS manifold. Our investigations reveal a phase transition in the applicability of this approach as a function of coupling to the environment—when the bath temperature and induced friction are sufficiently high, entanglement growth in individual trajectories is suppressed, and a low bond order description works for all time. This is a transition in the classical simulabiity of the open quantum system.

We believe that this transition is related to several other transitions in quantum dynamics that have been observed as a function of coupling the the environment or measurement, including the restriction of entanglement growth in random circuits with projective measurement, the quantum Zeno effect (and perhaps the KT transition in the spin-boson model^56–57^).

The implications of this result may be far-reaching. In the context of using the TDVP-Langevin equation to simulate open quantum systems, an efficient description for long times is possible for systems in the many-body quantum Zeno phase. Indeed, when a target system is in such a phase, there is no (asymptotic) advantage in using a quantum computer to simulate it. Since many chemical reactions of potential interest for quantum computation occur embedded in a dissipative aqueous environment, this is certainly a point worthy of consideration. An attractive feature of the TDVP Langevin approach to describing the dynamics is that it permits the use of the same semi-classical parametrization – the variational parameters – from early to late times and ultimately connects to the very late-time hydrodynamic limit.

Moreover, viewed from the perspective of a description of a quantum computational device or simulator, the transition into the many-body Zeno phase indicates transitions in the ability to solve quantum problems. While thresholds of noise for quantum error correction have been identified in the case of gate-based quantum computation, no such thresholds currently exist for adiabatic computation. It is intriguing to speculate that determining whether a putative adiabatic computational device is in its Zeno phase or not might provide similar bounds on performance^57,58^.

We envisage a number of ways in which this work might be developed. Extending the approach to local observables in closed quantum systems presents some exciting possibilities. In this case the bath would refer to other elements of the system itself and its properties self-consistently determined through the evolution^26,59^, Such a description has the promise of connecting early-time semi-classical descriptions to late-time hydrodynamics and thermalisation. Exploring the Fokker-Planck formulation of the TDVP-Langevin equation would bring a complementary perspective to our analysis^61–62^.

The accurate description of a quantum system from early to late times is generally not possible because of growing entanglement. However, coupling to the environment can limit this growth and render this achievable. This work has coordinated physical insights from several different perspectives to develop such a numerical scheme. We hope both that the algorithm itself will prove useful and that it will inspire further insights.

## Supplementary Information

Supplementary Information.

## Data Availability

The datasets generated and analysed in the current study are available in the UCL data repository at https://rdr.ucl.ac.uk/articles/dataset/All_data_for_phase_transitions_in_the_classical_simulability_of_open_quantum_systems_/22732289. Code used to produce this data is available at https://github.com/AndrewHallam/Langevin.
