# Supplementary material for: Phase transitions in the classical simulability of open quantum systems
Source: Sci Rep. 2023 May 31;13:8866. doi: 10.1038/s41598-023-35336-9 (PMC10232539; doi:10.1038/s41598-023-35336-9)
Supplement: Supplementary file 1 — Supplementary Information. [file 41598_2023_35336_MOESM1_ESM.pdf]

Supplementary Materials: Phase Transitions in the Classical Simulability of Open Quantum Systems

F. Azad,[1] A. Hallam,[2] J. Morley,[1] and A. G. Green[1]

[1]*London Centre for Nanotechnology, University College London, Gordon St., London, WC1H 0AH, United Kingdom*

[2]*School of Physics and Astronomy, University of Leeds, Leeds LS2 9JT United Kingdom*

(Dated: April 14, 2023)

In this supplementary we give further details of the derivation of the TDVP-Langevin equation and its numerical implementation, and present additional data showing the many-body Zeno transition. We begin in Sec. I with a demonstration that a thermal distribution over the variational manifold is a fixed point of the TDVP-Langevin equation. Next, in Sec. II we give both a heuristic and MPS path integral derivation of the TDVP-Langevin equation. Sec. III gives some more details of the numerical implementation. Additional data shows the many-body Zeno transition along different cuts through the temperature-friction parameter space are shown in Sec. IV. These data are presented in a form similar to Fig. 1 with the transition studied for fixed γ and γT. Finally in Sec. V we present details of how our data were rescaled in order to identify scaling collapse.

I. HAAR AVERAGING ON THE MPS MANIFOLD

In the left canonical form, MPS of left bond order D and local Hilbert space dimension D are given by $SU(dD)$ isometries[1]. In the case of a finite chain of length L, the left bond order at the nth site $D_n = d^n$ up to the maximum bond order at site $n = \log_d D_{\max}$. The bond order remains $D_{\max}$ up to site $L - 1 + \log_d D_{\max}$ beyond which it reduces as $D_n = d^{(n-L+1)}$.

The thermal expectation of an operator can be calculated by a Boltzmann-weighted Haar average over this variational manifold. The average of an operator $\hat{O}$ is given by

$$\langle\langle \hat{O} \rangle\rangle = \frac{\int \prod_n DA_n \langle\phi|\hat{O}|\phi\rangle \exp\left[-\beta\langle\phi|\hat{H}|\phi\rangle\right]}{\int \prod_n DA_n \exp\left[-\beta\langle\phi|\hat{H}|\phi\rangle\right]}. \quad (1)$$

The expectation of the Hamiltonian $\langle\phi|\hat{H}|\phi\rangle$ and the operator $\langle\phi|\hat{O}|\phi\rangle$ are calculated by usual MPS techniques. In practice, we carry out the integrals by by sampling a Haar random distribution of isometric MPS tensors; $A^{\sigma}_{ij} \equiv U_{i\otimes\sigma, j\otimes 1} \in SU(dD_n)$. These are obtained by a QR decomposition of a tensor with elements drawn randomly from a normal distribution.

Fig. 1 shows this Boltzmann-weighted Haar distribution as a function of energy at different temperatures. These thermal distributions are fixed-points of the dynamics described by Eq.(1)

II. DERIVING THE TDVP LANGEVIN EQUATION

Here we outline how the MPS TDVP-Langevin equation can be obtained. We follow two separate routes: a heuristic route using a solution of the Schrödinger equation for the bath and system, and constructing a Langevin limit of the Keldysh path integral constructed over MPS.

Bath Model: We model the bath as a collection of independent non-interacting harmonic oscillators. These are coupled to local system operators $\hat{F}_n$ at site n by their displacements. The Hamiltonian for the bath and its coupling to the system are given by

$$\hat{H}_{bath} = \sum_n \sum_\alpha \hbar\omega_\alpha \left(\hat{a}^\dagger_{n,\alpha}\hat{a}_{n,\alpha} + 1/2\right),$$
$$\hat{H}_I = -\sum_n \sum_\alpha \lambda_\alpha \left(\hat{a}^\dagger_{n,\alpha} + \hat{a}_{n,\alpha}\right)\hat{F}_n, \quad (2)$$

respectively. The index α labels the different oscillator modes at the site n. The distribution and temperature of the oscillator modes is assumed to be the same at each site. Moreover, we assume no back reaction of the system on the bath, so that the bath distribution remains equilibrium. This is a subtle assumption – the bath must be non-linear in order to thermalise energy absorbed from the system, but these non-linearities must operate on timescales such that the bath's effect on the system is the same as independent oscillators. The assumptions are standard - but non-trivial - and permit the simple manipulations that follow.

A heuristic derivation of the TDVP Langevin equation can be made in the spirit of the Frenkel principle for deriving the TDVP equations [2]. The state of the system and bath is parametrized as $|\psi(\mathbf{z})\rangle \otimes_{\mathbf{n},\alpha} |\phi_{\mathbf{n},\alpha}\rangle$ where $\mathbf{z}$ corresponds to some set of variational parameters of the system and $\phi_{n,\alpha}$ are coherent state parameters of the α-oscillator on site n. The time derivative of the wavefunction in the Schrödinger equation for the system and bath is expanded in a chain rule over the system and bath parameters:

$$\dot{\mathbf{z}}|\partial_{z_i}\psi\rangle \otimes_{n,\alpha} |\phi_{n,\alpha}\rangle + |\psi\rangle \sum_{m,\beta} \dot{\phi}_{m,\beta}\partial_{\phi_{m,\beta}} \left(\otimes_{n,\alpha}|\phi_{n,\alpha}\rangle\right)$$
$$\approx -i\hat{H}|\psi\rangle \otimes_{n,\alpha} |\phi_\alpha\rangle,$$

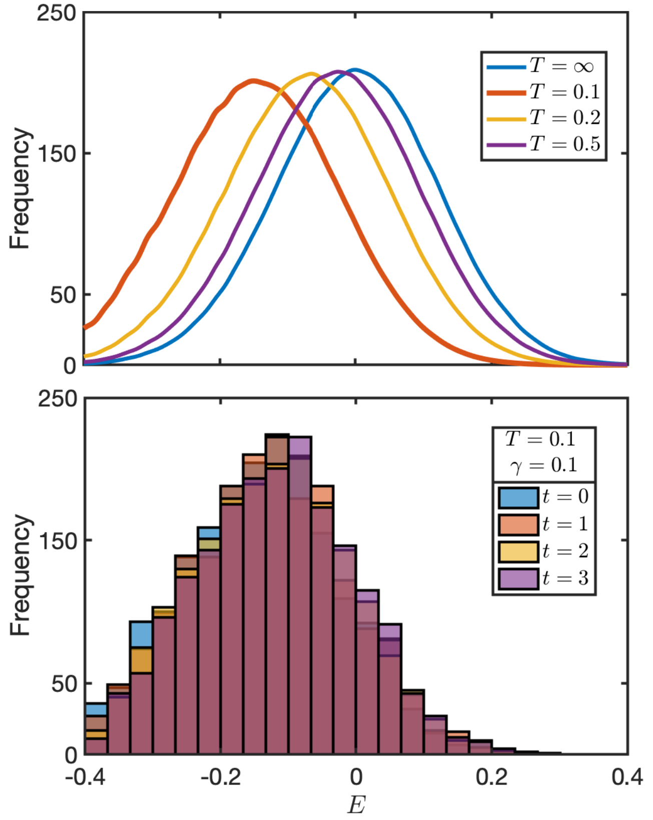

Supplementary Figure 1. *Density of occupied states at finite-temperature for a length 15 chain at bond order* Dmax $= 2$: a) The density of occupied states is computed for the Hamiltonian Eq.(2) with $J = 1$ $g = -1.05$, and $h = 0.5$ from a sample of Haar random distributed isometric MPS initial states. At $T = \infty$ — an unweighted Haar average — the majority of the states are in the middle of the spectrum and are highly entangled. A Boltzmann weight shifts this distribution to lower energy. Low-entanglement states are a subset of measure zero in the thermodynamic limit. b) The Boltzmann-weighted Haar average is a fixed point of the TDVP-Langevin equation. Here we compare distributions obtained after evolving with the Hamiltonian for $t = 1$, 2, and 3, with $\gamma = 0.1$ and $T = 0.1$. The distribution for $T = 0.1$ is shown in bold in a), we see the Langevin evolution preserves this distribution.

where the inequality is because the dynamics might take the state outside of the variational manifold. Taking an inner product with $\langle \partial_{z_i}\psi| \otimes_{n,\alpha} \langle \phi_{n,\alpha}|$ allows us to obtain an equation of motion for the system in the presence of the bath, and an inner product with $\langle \psi| \; \partial_{\phi_{o,\gamma}} (\otimes_{n,\alpha} \langle \phi_{n,\alpha}|)$ allows us to obtain an equation of motion for the bath in the presence of the system:

$$
\begin{aligned}
& i\langle \partial_{z_i}\psi|\partial_{z_j}\psi\rangle \dot{z}_j = \langle \partial_{z_i}\psi|\hat{H}_S|\psi\rangle \\
& + \sum_n \langle \partial_{z_i}\psi|\hat{F}_n|\psi\rangle \sum_\alpha \tilde{\lambda}_\alpha(\bar{\phi}_{n,\alpha}(t) + \phi_{n,\alpha}(t)) \\
& i\dot{\phi}_{n,\alpha} = \omega_\alpha + \lambda_\alpha \langle \psi|\hat{F}_n|\psi\rangle
\end{aligned}
\quad (3)
$$

The equalities are attained since the inner products with the tangent vectors project the Hamiltonian evolution back onto the variational manifold. These equations correspond to the usual TDVP equations with addition of coupling between the system and bath.

The remaining steps involve integrating the equation of motion for the bath degrees of freedom and substituting back into the equation of motion for the system. The formal solution of the equation of motion of the bath degrees of freedom is

$$
\phi_{n,\alpha}(t) = \phi_{n,\alpha}(0)e^{-i\omega_\alpha t} - \int_0^\infty D^R(t'-t)\langle \psi|\hat{F}_n|\psi\rangle dt'
$$

where we have identified the retarded bath correlator $D^R(t'-t) = -i\Theta(t-t')\langle \hat{a}^\dagger(t')\hat{a}(t)\rangle = ie^{i\omega(t'-t)}$. The assumptions of no back reaction of the system on the bath are taken account of by treating the terms $\eta_n(t) = \sum_\alpha \phi_{n,\alpha}(0)e^{-i\omega_\alpha t}$ as a stochastic random field with variance appropriate to the thermal distribution. After further identifying $\partial_t\Gamma(t) = D^R(t)$ the system of equation of motion can be put in the form

$$
i\langle \partial_{A_i}\psi|\partial_{A_j}\psi\rangle \dot{A}_j = \langle \partial_{A_i}\psi|\hat{H}_S|\psi\rangle + \sum_n \langle \partial_{A_i}\psi|\hat{F}_n|\psi\rangle \left(\int dt' \Gamma(t-t') \langle \partial_{A_j}\psi|\hat{F}_n|\psi\rangle \dot{A}_j + \eta(t) \right) \quad (4)
$$

the Markovian limit of which recovers Eq.(1).

A formal derivation from the Keldysh path integral can also be made. We follow the approach described in Ref.[3–5] constructing a Keldysh path integral for the density matrix and integrate out the bath in an appropriate limit to obtain a Langevin equation. Our main modification is to construct the path integral over MPS following Ref.[6].

The initial density matrix is assumed to factorise into density matrices for the system and bath as $\hat{\rho} \otimes \hat{\rho}_{bath}$ - the bath being in thermal equilibrium and its distribution assumed to be unchanged in time. This evolves to $\bar{T}e^{-i\int_0^t dt'(\hat{H}+\hat{H}_{int}+\hat{H}_{bath})}\hat{\rho} \otimes \hat{\rho}_{bath} T e^{-i\int_0^t dt'(\hat{H}+\hat{H}_{int}+\hat{H}_{bath})}$ at time t. Construction of the Keldysh path integral proceeds by dividing up the

time-ordered (T) and anti-time-ordered ($\bar{T}$) exponentials into many infinitesimal evolutions and inserting resolutions of the identity over coherent state variables for the bath and using a Haar measure over MPS states for the system[6]:

$$\mathbf{1} = \int \prod_n d\bar{\phi}_n(t) d\phi_n(t) e^{-\sum_n \bar{\phi}_n \phi_n} \otimes_n |\bar{\phi}_n\rangle\langle\phi_n|$$
$$\mathbf{1} = \int DA(t) |A\rangle\langle A|$$

Here DA symbolises an integral over all tensors in the MPS chain using the Haar measure introduced in Ref.[6]. A Keldysh rotation transforms from the fields on the time-ordered contour (+) and the anti time-ordered contour (−) to the sum and difference between them $A^{\pm} = A^{cl} \pm A^q$, $\phi^{\pm} = \phi^{cl} \pm \phi^q$, known as the classical and quantum components of the quantum field.

These manipulations give the following path integral for the evolution kernel of the density matrix:

$$K(t) = \int DAD(\bar{\phi}, \phi) e^{S[A^c, A^q, \phi^c, \phi^q]}$$
$$S = S[A^{cl} + A^q] - S[A^{cl} - A^q] + \int dt \left[\sum_{n,\alpha} \lambda_\alpha \left(F_n^q, F_n^{cl}\right) \begin{pmatrix} \phi^{cl}_{n,\alpha} + \bar{\phi}^{cl}_{n,\alpha} \\ \phi^{q}_{n,\alpha} + \bar{\phi}^{q}_{n,\alpha} \end{pmatrix} \right]$$
$$+ \int dt\, dt' \left[\sum_{n,\alpha} \left(\bar{\phi}^{cl}_{n,\alpha}(t), \bar{\phi}^{q}_{n,\alpha}(t)\right) \begin{pmatrix} 0 & [D^A_\alpha]^{-1}(t-t') \\ [D^R_\alpha]^{-1}(t-t') & [D^{-1}_\alpha]^K(t-t') \end{pmatrix} (t-t') \begin{pmatrix} \phi^{cl}_{n,\alpha}(t') \\ \phi^{q}_{n,\alpha}(t') \end{pmatrix} \right]$$

where D^A_α, D^R_α and D^K_α are the advanced, retarded and Keldysh components of the bath Greens function. Consistent with our assumption of a thermal equilibrium bath and no back reaction, they are related by the fluctuation dissipation relation: $D^K_\alpha(\omega) = \coth(\omega/2T)[D^R_\alpha(\omega) - D^A_\alpha(\omega)]$ with $D^{R(A)} = 1/(\omega \pm i\delta)$. $S[A]$ is the action of the system in the absence of coupling to the bath.

The simple quadratic form of the bath action follows from our assumptions and modelling of its effects as independent harmonic oscillators. It enables one to integrate out the bath and, depending upon timescales[**?**], to construct either a Lindblad or Langevin limit. We construct the latter limit in three steps. First the bath degrees of freedom are integrated out. The resulting dissipative contribution to the action has cross terms between classical and quantum components of the expectations of $\hat{F}$, and a term quadratic in the quantum component,

$$S_{diss} = \int dt\, dt' \sum_n \left(F_n^{cl}, F_n^q\right)(t) \begin{pmatrix} 0 & D^A(t-t') \\ D^R(t-t') & D^K(t-t') \end{pmatrix} \begin{pmatrix} F_n^{cl} \\ F_{n,}^q \end{pmatrix} (t'),$$

where the bath propagators without indices indicate a sum over all modes, for example $D^R = \sum_\alpha \lambda_\alpha^2 D^R_\alpha$. The quadratic term in F^q is decoupled with a Hubbard-Stratonovich field $\eta(t)$ that ultimately will play the role of the stochastic noise field in Eq.(1). The final trick to bring this integral to the Langevin form is to Taylor expand the action to linear order in the quantum fields, A^q. The result is a path integral over the MPS tensors A^q and A^{cl} and the noise field η:

$$K(t) = \int DA^q DA^{cl} D\eta e^{iS[A^c, A^q, \eta]}$$
$$S = \int dt \sum_n A^q_n(t) \underbrace{\left[2\delta S[A^{cl}]/\delta A^{cl}_n(t) + 2\sum_m \partial F^{cl}_m/\partial A^{cl}_n(t) \left(\int dt'\; D^R(t-t') F^{cl}_m(t') + \eta_m(t) \right) \right]}_{\text{Eq.(1)}}$$
$$\left. - \int dt\, dt' \sum_n \eta_n(t) [D^K]^{-1}(t-t') \eta_n(t') \right].$$

This is equivalent to the TDVP Langevin of Supplementary Eq.(4); the quantum field A^q plays the role of a Lagrange multiplier that imposes Supplementary Eq.(4) and the remaining term gives the bath correlations. The tensor indices have been suppressed for clarity in this expression.

To make the comparison with the TDVP-Langevin equation, note that $\delta S[A^{cl}]/\delta A^{cl}_n(t) = 0$ recovers the usual TDVP equations for matrix product states. The additional terms correspond to the dissipative effects of

the bath. These terms are non-local in the chain indices n and m, despite our model of local independent baths. This is due to the potential long-ranged entanglement of the matrix product state. The long-range effects of the noise term reflect those already found in the usual TDVP equations, since the noise term arises from a random local potential. The non-locality of the friction term is more problematic and some insight is required to implement it efficiently.

III. IMPLEMENTING THE TDVP LANGEVIN EQUATION

Here we outline how the TDVP-Langevin equation, Eq.(1), can be implemented numerically for MPS. This equation comprises three parts. The first is the closed-system TDVP equation. This is implemented by standard means. The second is the random noise induced by the environment. This is essentially a time-dependent Hamiltonian term. We integrate it in a Stratonovich scheme. The final part is friction. Even in the Markovian limit, this term is generally spatially non-local — however, a significant simplification can be achieved by working with purely local operators.

The TDVP equations for MPS can be written n the form

$$\begin{aligned} \langle \partial_i \psi | \partial_j \psi \rangle \dot{X}_j &= -i \langle \partial_i \psi | \hat{H} | \psi \rangle \\ \Rightarrow \quad \langle \partial_{A_n} \psi | \partial_{A_m} \psi \rangle \dot{A}_m &= -i \langle \partial_{A_n} \psi | \hat{H} | \psi \rangle \end{aligned} \tag{5}$$

where we have suppressed the tensor indices of A for clarity, retaining only the site index. The solution of this equation is well established. A judicious choice of gauge fixing for the tangent vectors to the MPS manifold puts the Gramm matrix $\langle \partial_{A_n} \psi | \partial_{A_m} \psi \rangle$ in a diagonal form. Various algorithms for evaluating the TDVP equations for finite systems exist, in this report we have used a modification of the method introduced in [7]. A single time-step of the algorithm is achieved by sweeping through the system from right to left and applying a unitary rotation to the local variables on each site $A_n(t + \delta t) = e^{iH_{eff}} A_n(t)$, followed by repeating this process by sweeping from left to right.

The Noise contribution to the TDVP Langevin equation for MPS can be written in the form

$$\begin{aligned} & -i \sum_m \langle \partial_i \psi | \hat{F}_m | \psi \rangle \eta(t) \\ & \Rightarrow -i \sum_m \langle \partial_{A_n} \psi | \hat{F}_m | \psi \rangle \eta_m(t) \end{aligned} \tag{6}$$

This evidently takes the same form as the right hand side of Supplementary Eq.(5) and no substantial modification to the TDVP algorithm is required. At the beginning of each timestep, $\eta_m(t)$ is chosen by sampling from a normal distribution with mean zero and variance $2\delta t \gamma T$.

Friction: The friction term can be written in the Markovian limit and in terms of MPS tensors as follows:

$$-i \sum_m \gamma \frac{\langle \psi | \hat{F}_m | \psi \rangle}{dt} \langle \partial_i \psi | \hat{F}_m | \psi \rangle, \tag{7}$$

where

$$\frac{\langle \psi | \hat{F}_m | \psi \rangle}{dt} = \langle \psi | \hat{F}_m | \partial_{A_n} \psi \rangle \dot{A}_n + \langle \partial_{\bar{A}_n} \psi | \hat{F}_m | \psi \rangle \dot{\bar{A}}_n. \tag{8}$$

Supplementary Eq.(8) can be evaluated by substituting in Supplementary Eq.(5) introducing the poisson bracket notation, $\{O_1, O_2\} = i \langle \psi | \hat{O}_1 | \partial_{A_n} \psi \rangle \langle \partial_{\bar{A}_n} \psi | \hat{O}_2 | \psi \rangle - i \langle \psi | \hat{O}_2 | \partial_{A_n} \psi \rangle \langle \partial_{\bar{A}_n} \psi | \hat{O}_1 | \psi \rangle$,

$$\begin{aligned} & (\delta_{m,n} + \sum_n \gamma \{F_m, F_n\}) \frac{\langle \psi | \hat{F}_n | \psi \rangle}{dt} = \{F_m, H\} + \sum_n \{F_m, F_n\} \eta_n(t) \\ & \rightarrow \frac{\langle \psi | \hat{F}_n | \psi \rangle}{dt} = (\mathbb{I} + \gamma \mathbb{F})^{-1} \left(\{F_m, H\} + \sum_n \{F_m, F_n\} \eta_n(t) \right) \end{aligned} \tag{9}$$

where $\mathbb{F}_{ij} = \{F_i, F_j\}$. Evaluating Supplementary Eq.(9) exactly for arbitrary operators $\hat{F}_n$ is quite numerically inefficient, scaling quadratically in the number of noise operators. Moreover, it is inconsistent with the site by site sweep algorithm introduced above for the TDVP equations. Fortunately, Supplementary Eq.(9) is substantially simplified in the case of single-site, local noise fields. For local fields $\{F_i, F_j\}$ is only non-zero provided the two operators are located on the same site of the system and so $\mathbb{F}$ becomes a simple, block diagonal matrix.

The modified TDVP algorithm therefore works as follows: Before each sweep through the system $\eta_m(t)$ is sampled from a normal distribution with mean 0 and variance $2\delta t \gamma T$. The Hamiltonian and noise terms are then used to calculate $\frac{\langle \psi | \hat{F}_n | \psi \rangle}{dt}$ for all noise operators F_n using Supplementary Eq.(9). The noise and friction terms combine with the Hamiltonian using Supplementary Eq.(5) and the state is evolved using the standard TDVP algorithm.

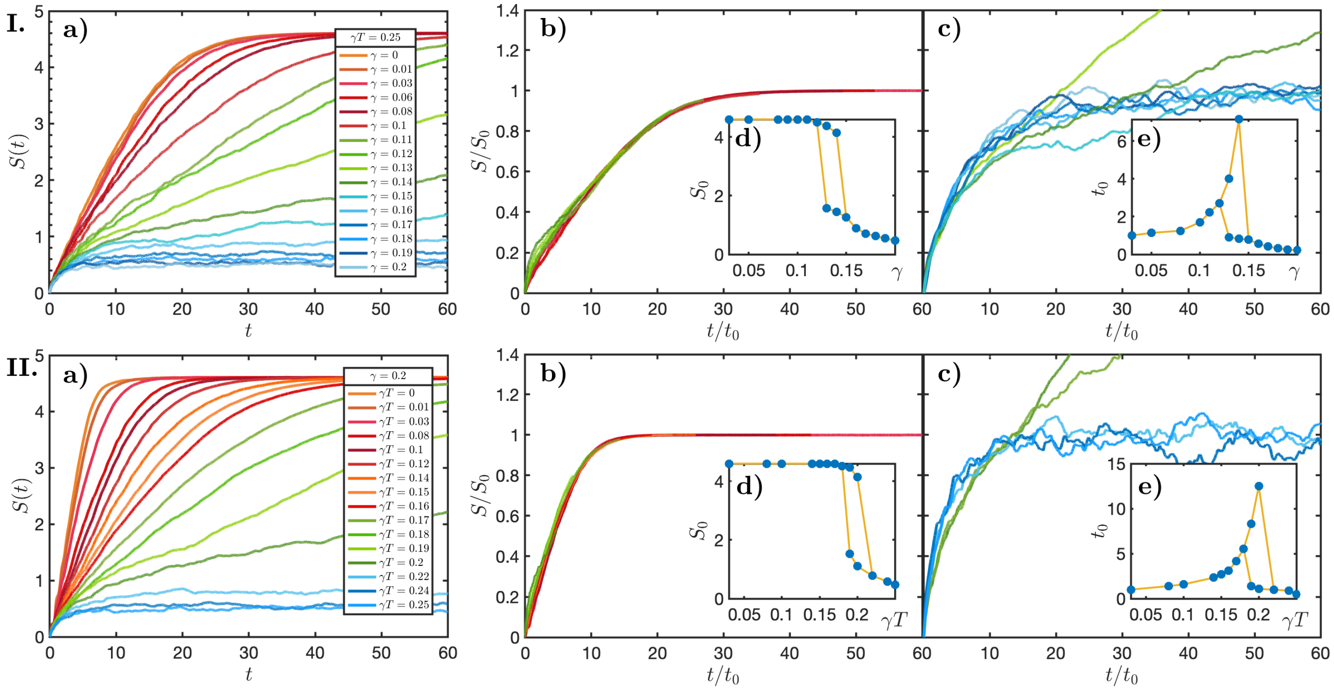

Supplementary Figure 2. *Many-body Zeno transition in the* $\gamma - T$ *plane.* These results extend Fig. 1 of the main text a) Further values for the von Neumann entropy as a function of time are shown for averaged trajectories at different coupling strengths. In I. we have kept noise fixed with $\gamma T = 0.25$, while in II. we keep friction fixed with $\gamma = 0.2$. These simulations have bond dimension kept at $D = 128$ and serve as the reference case for the comparisons made against lower bond dimension simulations. Curves b) and c) show the data collapse from obtained by rescaling the amplitude by S_0 and the timescale by t_0 before and after the many-body Zeno transition, respectively following the procedure described in the text. A few curves near the transition show different early and late time behaviour leading to the apparent hysteresis in the plots of the extracted S_0 and t_0 shown in the insets d) and e) respectively.

IV. MANY-BODY ZENO TRANSITION AS A FUNCTION OF NOISE AND FRICTION

In Fig. 1 of the main text we present results for the many-body Zeno transition at fixed temperature T as a function of friction γ. Here we present results for the dependence of the many-body Zeno transition as a broader function of coupling to and temperature of the environment. Previous studies of transitions in monitored Hamiltonian systems have not captured this broader dependence, since they were based upon unravellings of the Lindblad equation which has an infinite temperature fixed point.

In Supplementary Fig. 2 we show results for transitions as a function of friction γ at constant noise $\gamma T = 0.25$, and as a function of noise γT at constant friction $\gamma = 0.2$. The overall message is similar to that presented in Fig. 1 of the main text: above a threshold noise or friction, the system undergoes a transition to a many-body Zeno phase. Moreover, a good collapse of the data above and below the transition is obtained by rescaling the entanglement by S_0 and the timescale by t_0 shown in the insets I and II d), and I and II e), respectively.

A. A Pre-Many-Body Zeno Phase.

Supplementary Fig. 2 reveals a new feature not found as a function of γ at fixed T. A few curves around the transition values – $\gamma \approx 0.12 - 0.14$ at fixed $\gamma T = 0.25$ and $\gamma T \approx 0.19 - 0.20$ at fixed $\gamma = 0.2$ – show an early-time behaviour consistent with the many-body Zeno phase, before crossing over to a late time behaviour that does not. The insets I d) and e), and II d) and e) in Supplementary Fig. 2 demonstrate this hysteresis in the rescaling factors S_0 and t_0 obtained by fitting the early-time and late time behaviour separately [see Sec.V for more details about these fits]. Supplementary Fig. 3 overlays the hysteretic rescaling data of the insets I d) and II d) from Supplementary Fig. 2 over the saturation entanglement curves of Fig. 3 from the main paper. Supplementary Fig. 5 b) and d) illustrate this difference in early- and late-time behaviour for particularly individual trajectories. Our interpretation of these data is that the many-body Zeno transition confines trajectories to a low entanglement region of the variational manifold. Near the transition certain trajectories may have a very long dwell-time in this reduced entanglement phase before ultimately being kicked into the higher entanglement region. This might be termed a pre-Zeno plateau.

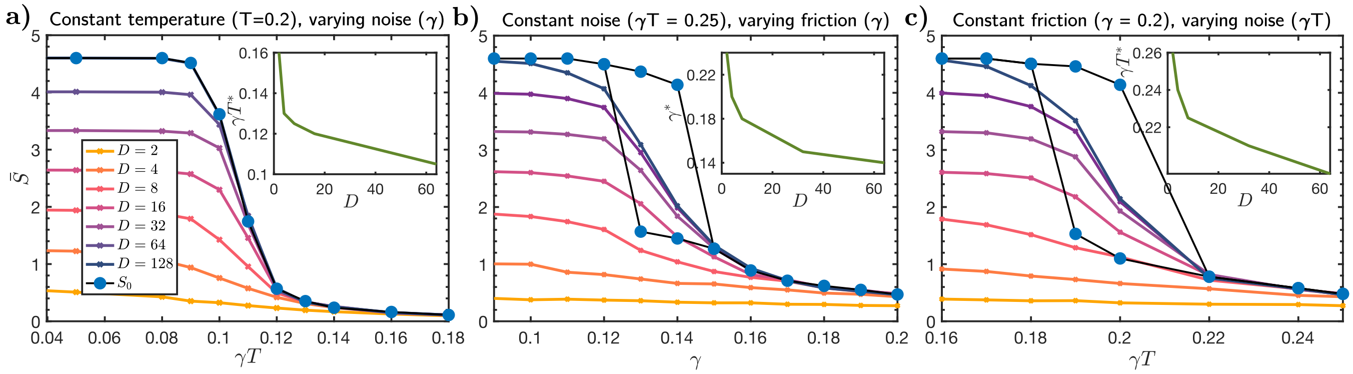

Supplementary Figure 3. *Evolution of Saturation Entanglement at Finite Temperature:* Here we have shown Fig. 3 from the main text with curves for S_0 overlayed. a) In the case where T is kept fixed, the curve for S_0 is exactly the $D = 128$ simulation since clear entanglement saturation occurs. In b) and c) we have overlayed the curves for S_0 as seen in Supplementary Fig. 2d. Near to the many-body Zeno transition a few curves show different early- and late-time behaviour leading to an apparent hysteresis in S_0.

V. EXTRACTING SCALING COEFFICIENTS

The essence of our scaling collapse shown in Fig. 1 and Supplementary Fig. 2 is to rescale entanglement by a factor of S_0 and timescales by a factor of t_0. We identify these factors by fitting to two analytical forms: either $S(t) = S_0 \tanh(t/t_0)$ or $S_0(1 - \exp(-1.4\, t/t_0))$ (the factor of 1.4 in the latter gives approximately the same fitted t_0 with either function). These functions are heuristics used to extract our scaling. In principle such a fit is not necessary, but we find it a good way to obtain our scaling. Moreover, the fits are actually rather good. Supplementary Fig. 4 shows a typical fit to $S(t)$ averaged over trajectories. For data at constant temperature shown in Fig. 2 in the main body of text, we find that the form $S(t) = S_0 \tanh(t/t_0)$ gives a reasonable fit both above and below the many-body Zeno transition. In the cases of constant noise and constant friction, we find a slightly better fit using $S_0(1-\exp(-1.4\, t/t_0))$ in the many-body Zeno phase Similar fits (to $\tanh(t/t_0)$) have been used in random unitary circuits [8, 9]. This can explain the quality of fit in the infinite-temperature/zero-friction case, with $\gamma T > 0.15$, where the dynamics are akin to random unitary circuit.

Typical Fits: In Supplementary Fig. 4b), we show a typical fitting to $S(t) = S_0 \tanh(t/t_0)$ for the case where $T = 0.2$ and $\gamma T = 0.1$. This trajectory is in the many-body Zeno phase as can be seen by the value of $S_0 = \bar{S} = 3.6$, which is less than the maximum value of 4.6 found in these simulations.

Pre-Zeno Fits: As mentioned above, in the case of many-body Zeno transitions at constant noise or constant friction, we find that trajectories near the transition display early-time behaviour typical of the many-body Zeno phase crossing at early times over to a late-time behaviour typical of trajectories in which the environment does not restrict the entanglement growth. Supplementary Fig. 5 shows how we treat such trajectories. Panel b) shows averaged trajectories at $\gamma = 0.19$, $\gamma T = 0.25$ and $\gamma = 0.18$, $\gamma T = 0.25$ with fits to $S(t) = S_0 \tanh(t/t_0)$ at late times and to $S_0(1 - \exp(-1.4\, t/t_0))$ early times. Panel a) shows 70 trajectories that are averaged to obtain the $\gamma = 0.19$, $\gamma T = 0.25$ results. It is from these individual trajectories that the underlying behaviour is one of apparent saturation to a many-body Zeno phase before a random kick from the leaving the low entanglement region of the manifold at some random time.

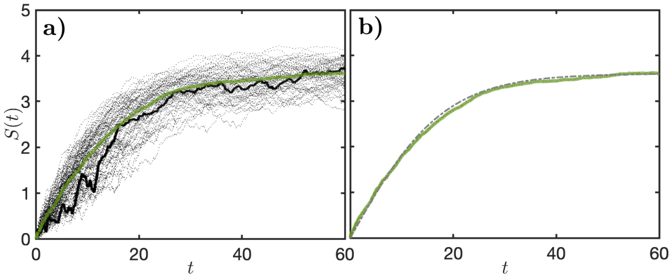

Supplementary Figure 4. *Scaling Fit to* $S(t)$: Here we show the fitting procedure used to determine S_0 and t_0 for our scaling collapse for the case fixed temperature and varying noise. a) Shows 70 trajectories for $T = 0.2$ and $\gamma T = 0.1$. For these parameters, the system is in the many-body Zeno phase. We highlighted a typical trajectory in bold and we have overlay the average of these trajectories as depicted in Fig. 1 in the main text. b) The dashed line shows the fitting $S(t) = S_0 \tanh(t/t_0)$ with $S_0 = \bar{S} = 3.6$ and $t_0 = 18$.

[1] Orús, R. A practical introduction to tensor networks: Matrix product states and projected entangled pair states. *Annals of physics* **349**, 117–158 (2014).

[2] Frenkel, J. *Wave mechanics, advanced general theory*, vol. 1 (Oxford, 1934).

[3] Kamenev, A. Keldysh and doi-peliti techniques for out-of-equilibrium systems. In *Strongly Correlated Fermions and Bosons in Low-Dimensional Disordered Systems*, 313–340 (Springer, 2002).

[4] Kamenev, A. *Field theory of non-equilibrium systems* (Cambridge University Press, 2011).

[5] Crowley, P. J. & Green, A. Anisotropic landau-lifshitz-gilbert models of dissipation in qubits. *Physical Review A* **94**, 062106 (2016).

[6] Green, A., Hooley, C., Keeling, J. & Simon, S. Feynman path integrals over entangled states. *arXiv preprint arXiv:1607.01778* (2016).

[7] Haegeman, J., Lubich, C., Oseledets, I., Vandereycken, B. & Verstraete, F. Unifying time evolution and optimization with matrix product states. *Physical Review B* **94**, 165116 (2016).

[8] Bera, A. & Singha Roy, S. Growth of genuine multipartite entanglement in random unitary circuits. *Phys. Rev. A* **102**, 062431 (2020). URL `https://link.aps.org/doi/10.1103/PhysRevA.102.062431`.

[9] Nahum, A., Vijay, S. & Haah, J. Operator spreading in random unitary circuits. *Physical Review X* **8**, 021014 (2018).

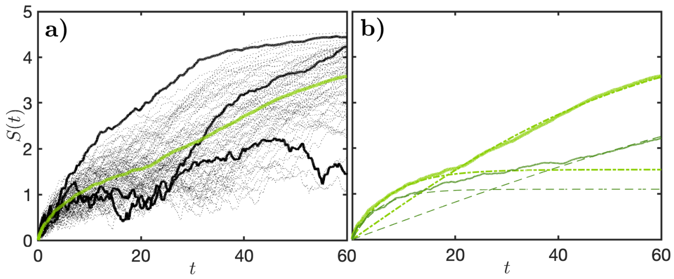

Supplementary Figure 5. *Scaling Fit for pre-Zeno Trajectories:* Here we show how scaling is determined for those trajectories near to the many-body Zeno transition that display different early- and late-time behaviour. a) 70 trajectories with $\gamma T = 0.14$ and $\gamma = 0.2$ together with the average trajectory shown in green. Individual trajectories show signs of saturation of entanglement which persists for a period of time before beginning to rise again. b) The average trajectory can be fit in two different ways at early and late times. Here we show these fits for trajectories $\gamma T = 0.14$ and $\gamma = 0.2$, and $\gamma T = 0.15$ and $\gamma = 0.2$ using $S_0(1 - \exp(-1.4\, t/t_0))$ for early times and $S(t) = S_0 \tanh(t/t_0)$ for later times.
